# Grand Challenges in Oral Infections and Microbes

**DOI:** 10.3389/froh.2020.00002

**Published:** 2020-06-09

**Authors:** Georgios N. Belibasakis

**Affiliations:** Division of Oral Diseases, Department of Dental Medicine, Karolinska Institutet, Huddinge, Sweden

**Keywords:** oral infections, oral microbes, oral health, oral disease, dentistry, biofilms, oral microbiology and immunology, oral microbial ecology

Oral diseases are widespread among the global population. It is estimated that, as of the middle of the previous decade, 3.5 billion people were living with untreated oral conditions [1]. Apart from the morbidities caused by these diseases on an individual level, they pose a significant public health challenge for national health systems. The most common oral diseases are of microbial etiology, and include dental caries, odontogenic infections, periodontitis, peri-implantitis, and stomatitis [2].

Studies spanning over a century have attempted to decipher the microbes involved in these oral infections by microscopy, culture, and molecular techniques. We now understand that they are largely endogenous polymicrobial infections. In other terms, they are caused by biofilms formed by multiple and varying resident microorganisms that may, under certain conditions, overgrow to act as opportunistic pathogens. We have gathered considerable scientific information on the microbial etiology and pathogenesis of oral infectious diseases, which is often highlighted in specialized fora [3, 4]. Despite the great scientific progress made in their understanding, oral infections still pose an important prevention and therapeutic challenge for individual patients, dental clinicians, and national health systems alike [2].

Current research trends dictate the use of state-of-the-art high-throughput sequencing technologies for characterizing the oral microbiome and its dysbiotic changes that lead from health and disease [5]. The study of the oral microbiome with open-ended methods allows the discovery or verification of previously unknown, uncultivated, or unsuspected microorganisms in oral infections. It also enables the individual quantification and simultaneous study of interrelationships between microorganisms in a given sample [6]. The term “oral microbiome” is often used interchangeably with “oral microbiota,” yet the former implies the collection of genes present in the latter community of microorganisms. The oral microbiome is an integral residential component of the oral cavity and is considered to be the second more diverse regional microbiome of the human body [7]. Stability of the microbiomes results from a dynamic, yet balanced, interaction of microbes with each other, and with their host, in their micro-ecological niche. Environmental pressures that exceed thresholds associated this homeostatic balance will challenge the competitiveness of the resident microbiota leading to dysbiosis and imminent disease. Paradoxically, the diversity of the focal microbiome appears to increase rather than decrease in given oral infections, such as periodontal disease [8–10]. Therefore, measuring the stability of the oral microbiome could be an important utility for clinical prognosis [11] and diagnosis [12]. Achieving the stability linked to a clinically healthy status should be a key consideration for future preventive and therapeutic strategies [13]. Identification of the biological properties that confer this stability is important for preventing the dysbiotic microbial shifts. Acquiring and maintaining a “healthy oral microbiome” is a promising field open for further investigations [14]. Accordingly, for long-term efficacy, research efforts should largely be placed on prevention rather than treatment strategies. Our current understanding of oral infections and their associated oral microbiota urges us to reconsider our clinical thinking by embracing more “ecological” approaches for prevention and treatment. In other words, we should strive for preventing or reversing the biological, behavioral, or environmental factors that favor the overgrowth of opportunistic pathogens and the consequent dysbiotic changes that lead to disease [13].

While metagenomics have been instrumental in taxonomically characterizing the oral microbiome, the added dimensions of metatransctriptomics, metaproteomics, and metabolomics enable the classification of the oral microbiota according to biosynthetic, functional, and metabolic properties. These may not necessarily overlap with phylogenetic taxonomy, but the collective assessment of multiple related biological units (genes, gene expression, proteins, metabolites) may enable us to better understand the dynamics of dysbiosis in oral infections. Among the biggest challenges in combined “omics” are data clarity with reduced background noise, reproducibility among different study cohorts, computational integration of different data layers, and meaningful data interpretation for oral disease [6]. Multifaceted data-driven approaches are needed in oral healthcare research. They enable integration of diverse individual characteristics, in order to be able to stratify patients according to individual risk groups and oral healthcare requirements. Using, for instance, microbiological parameters (e.g., stability and dynamics of microbiome or metabolic pathways), adding the patients' risk factors (e.g., genetic, environmental, and behavioral), and channeling the data through supervised and unsupervised learning systems may help identify and manage particularly susceptible individuals or populations. This will customize treatment planning and improve the treatment outcome, moving us away from decision-making based on experiential opinion or clinical presentation alone, in line with the concept of precision dentistry [15, 16].

The need for personalized monitoring and treatment in dental healthcare is now more evident than ever, and certain facets of our acquired knowledge on the oral microbiome can be well-utilized in this direction [16–18]. Continuously monitoring microbiological parameters, alone or in combination with immunological parameters, in a fast and cost-efficient manner before treatment or during maintenance will facilitate the applications of precision dentistry and personalized oral healthcare. Development of point-of-care devices for microbiological and immunological detection that would assist clinicians in the diagnostic, monitoring, and selection of treatment aspects are underway [19, 20].

One of the most contemporary threats to global health is the advent of antimicrobial resistances. Antibiotics for oral infections are prescribed for prophylactic or therapeutic purposes, but are most often based on professional experience or excessive precaution, rather than scientific evidence, susceptibility tests, or good practice guidelines. Common antibiotic prescriptions in dentistry exceed several other medical and allied health care provider specialties [21] and may not always be deemed necessary [22]. Antibiotics may cause ecological disturbances of the resident microbiota at various sites of the body, even ones distant to the focal infection. They can also induce the selection of resistant strains in the microbiome. Antibiotic-resistant strains can be found in individuals not recently exposed to antibiotics, and among commensal bacterial species, not only pathogens. This has led to a paradigm shift of how resistances are transferred across the oral microbiome. We now understand that the oral microbiome may act as a general reservoir for the carriage of antibiotic resistances, leading to the concept of the “oral resistome” [23]. In this light, significant research efforts should be placed on preventing or curtailing the unnecessary use of antibiotics for dental purposes, in line with the “One Health” concept that unifies human, public, and environmental health [24, 25]. In parallel, this raises the need for the discovery and implementation of alternative antimicrobial approaches in dentistry, including manufactured or natural antimicrobial agents [26, 27], photodynamic therapy [28], or concomitant use of adjunctive host modulating agents [29].

A joint forum of communication between basic and clinical research is imperative to address these emerging challenges of the oral domain in the years to come, for the collective benefit of the individual and the population. The Specialty Section of “Oral Infections and Microbes” of *Frontiers in Oral Health* aspires to become a representative forum for fulfilling this goal.

## Author Contributions

The author confirms being the sole contributor of this work and has approved it for publication.

## Conflict of Interest

The author declares that the research was conducted in the absence of any commercial or financial relationships that could be construed as a potential conflict of interest.
